# Comparative effectiveness of external therapies of traditional Chinese medicine and surgical treatments in pain management of postherpetic neuralgia: A protocol for a systematic review and network meta-analysis

**DOI:** 10.1097/MD.0000000000031517

**Published:** 2022-10-28

**Authors:** Zhengqi Pan, Shijie Huang, Tingting Ma, Rongli Yuan, Mengjing Wang, Rui Luo, Maogui Yu, Wuyu Li, Ao Zhang, Jie Wu

**Affiliations:** a Acupuncture and Tuina School, Chengdu University of Traditional Chinese Medicine, Chengdu, Sichuan Province, China; b Hospital of Chengdu University of Traditional Chinese Medicine, Chengdu University of Traditional Chinese Medicine, Chengdu, Sichuan Province, China; c School of Chinese Medicine, School of Integrated Chinese and Western Medicine, Nanjing University of Chinese Medicine, Nanjing, Jiangsu Province, China.

**Keywords:** external therapies of traditional Chinese medicine, network meta-analysis, pain alleviation, postherpetic neuralgia, randomized controlled trial, surgical treatments, systematic review

## Abstract

As the most common long-term complication of herpes zoster (HZ), postherpetic neuralgia (PHN) is characterized by chronic, persistent, and debilitating neuropathic pain. PHN seriously harms human health and currently becomes a topic of clinical importance. To date, the common methods of pain management in PHN include external therapies of traditional Chinese medicine and surgical treatments. However, there is no high-quality or direct evidence of their comparative effectiveness. This review aims to provide a network meta-analysis to compare the efficacy of external therapies of traditional Chinese medicine and surgical treatments in the pain management of PHN.

**Methods:**

Databases such as PubMed, Cochrane Central Register of Controlled Trials, EMBASE, China National Knowledge Infrastructure, China Biology Medicine Disc will be searched for relevant randomized controlled trials to obtain literatures on the treatment of PHN with external therapies of traditional Chinese medicine and surgical treatments, and clinical randomized controlled trials will be screened out from their inception to August 5, 2022. The participant intervention comparator outcomes of this study are as flowing: P, patients with PHN; I, external therapies of traditional Chinese medicine and surgical treatments; C, no treatment, pharmacological placebo, treatment as usual or sham acupuncture groups; O, primary outcome is pain intensity, and secondary outcomes are onset of pain relief time, quality of life, therapeutic effective rate and reverse effects. Cochrane Risk of Bias Tool will be used in assessing literature’s quality. Network meta-analyses will be conducted to generate estimates of comparative effectiveness of each intervention class and rankings of their effectiveness, in terms of pain management.

**Result:**

This systematic review and network meta-analysis will provide evidence of the efficacy of different therapeutic methods for pain management in PHN, to show which forms of therapy are more commonly used with higher effectiveness.

**Discussion:**

The results will systematically provide suggestions for medical practitioners to choose effective, time-saving and economical pain management method for PHN.

## 1. Introduction

Postherpetic neuralgia (PHN) is the main complication caused by Herpes Zoster (HZ). PHN is a long-lasting and difficult-to-treat disease characterized by chronic, persistent, and debilitating neuropathic pain as the main symptom.^[1]^ Moreover, patients with PHN often suffer from allodynia, hyperalgesia, and abnormal sensations within the affected dermatome or beyond the dermatomal margins. The current incidence of HZ in North America, Europe and the Asia-Pacific region ranges from 3 to 5/1000 person-years, of which 6% to 30% will develop PHN^[2]^; among HZ patients over 50 years old, 60% to 70% will develop PHN, and the incidence and treatment cost of PHN increase with age.^[2,3]^ PHN severely reduces the quality of life of patients and imposes a great economic burden on human society worldwide; therefore, it is important to find the rational and effective medical option for PHN.

Currently, surgical treatment is a new trend in the clinical strategy of PHN pain treatment, which includes minimal invasive surgery, nerve block, intrathecal drug injection, pulsed-radiofrequency.^[4,5]^ However, There are more or less problems with these treatments, such as limited efficacy, serious adverse reactions, and high cost.^[6]^ For example, the nerve block treatment often leads to some complications, such as hemorrhage, hematoma, pneumothorax, nerve injury, infection, etc.^[7,8]^ Patients with PHN are often frail and have multiple comorbidities; therefore, it becomes even more important to minimize the side effects of interventions.^[9]^

External therapies of traditional Chinese medicine such as acupuncture, moxibustion, acupoint injection, have been showing its significant therapeutic effects in pain management.^[10,11]^ In many countries such as China, Japan, and Italy, in addition to the above pharmacological interventions, external therapies of traditional Chinese medicine are widely applied in pain management of PHN.^[12–14]^ There are many clinical and experimental studies have reported the therapeutic effects of external therapies of traditional Chinese medicine in treating PHN; in clinical practice, external therapies of traditional Chinese medicine have been identified as an important method with promising results in relieving patient’s pain with tiny adverse reactions.^[15,16]^ External therapies of traditional Chinese medicine appears to be an attractive alternative to other intervention as a first-line treatment due to its comparatively fewer and less severe side-effects.

Despite both external therapies of traditional Chinese medicine and surgical treatments being commonly used in treating PHN, few studies have directly compared them and the previous systematic reviews only evaluated the treatment of PHN with external therapies of traditional Chinese medicine and surgical treatments separately. This study aims to compare the efficacy of external therapies of traditional Chinese medicine and surgical treatments in the pain management of PHN through a network meta-analysis; external therapies of traditional Chinese medicine and surgical treatments will be ranked in order to determine the most effective methods, which will provide evidence for choosing certain therapeutic strategies in the pain management of PHN.

## 2. Method

This systematic review protocol has been registered on PROSPERO with number CRD42022361896 (https://www.crd.york.ac.uk/PROSPERO/display_record.php?RecordID=361896). This protocol will be reported according to the Preferred Reporting Items for Systematic Reviews and Meta-Analyses protocols (PRISMA)^[17]^and this network meta-analysis will be conducted and reported in accordance with PRISMA extension version (PRISMA-NMA).^[18]^ We will also apply the International Society for Pharmacoeconomics and Outcomes Research Indirect Treatment Comparison/Network Meta-Analysis Study Questionnaire to Assess Relevance and Credibility to Inform Health Care Decision-Making to our study to aid the interpretation of clinicians and other healthcare decision-makers.^[19]^

### 2.1. Eligibility criteria

#### 2.1.1. Population.

Participants with PHN, regardless of age, sex or ethnicity, were included in this study. Referring to previous studies,^[20]^ PHN is defined as pain persisting for more than 1 month after the skin lesions caused by HZ have resolved, or any of the above criteria included into the literature.

#### 2.1.2. Interventions.

The systematic review will focus on interventions which are intended to relieve pain in patients with PHN using either external therapies of traditional Chinese medicine and surgical treatments; among which, the surgical treatments are derived from the first-line treatments in the recent PHN guidelines and consensus, including minimal invasive surgery, nerve block, intrathecal drug injection, pulsed-radiofrequency.^[5,21]^

#### 2.1.3. Comparators.

There are a number of types of comparator conditions which will be eligible for inclusion in the network of evidence. The types of control conditions may include no treatment, pharmacological placebo, treatment as usual or sham acupuncture groups. Furthermore, different types of external therapies of traditional Chinese medicine and surgical treatments may also be directly compared.

#### 2.1.4. Outcomes.

The primary outcome in this study will be pain intensity, which is measured by the Visual Analogue Scale, Numerical Rating Scale, as well as several other scales for measuring pain.

The secondary outcome will include onset of time for pain relief, quality of life, therapeutic effective rate and reverse effects.

#### 2.1.5. Study designs.

The systematic review will only include randomized controlled trials. These trials must have a sample size of at least 20 participants per condition and must involve interventions delivered for a minimum of 4 weeks. To reduce heterogeneity, crossover trials will be excluded. Studies making within-class comparisons only (e.g., ordinary acupuncture versus electroacupuncture) will also be excluded. Finally, studies must be reported in English or in Chinese.

### 2.2. Information sources and search strategy

We will search PubMed, Cochrane Central Register of Controlled Trials, EMBASE, China National Knowledge Infrastructure, China Biology Medicine Disc from inception to August 5, 2022 with the language restriction of English and Chinese. Randomized controlled trials that exhibited the effective therapies of PHN will be selected. The search keywords contain PHN (e.g., postherpetic neuralgia, herpetic pain), first-line surgical treatments for treating PHN^[4,22]^(e.g., minimal invasive surgery, nerve block, intrathecal drug injection, pulsed-radiofrequency), external therapies of traditional Chinese medicine (e.g., acupuncture, acupoint injection, electroacupuncture, moxibustion, cupping, tuina). The search strategy for PubMed is shown in Table 1, and other electronic databases will be searched with the same strategy.

**Table 1:** Search strategy for PubMed database.

Number	Search terms
#1	“ Postherpetic neuralgia “ [exploded MeSH] OR “ Postherpetic neuralgia “ [Title/Abstract] OR “ Post-herpetic neuralgia “ [Title/Abstract] OR “ Post herpetic neuralgia “ [Title/Abstract] OR “ PHN” [Title/Abstract] OR “ post-herpetic pain “ [Title/Abstract] OR “ post herpetic pain “ [Title/Abstract]
#2	“ Neuralgia “ [exploded MeSH] OR “ Pain “ [exploded MeSH] OR “ neuralgia “ [Title/Abstract] OR “ Pain “ [Title/Abstract]) AND (“ Herpes zoster” [exploded MeSH] OR “ Zoster “ [Title/Abstract] OR “ Shingles “ [Title/Abstract] OR “ Zona “ [Title/Abstract] OR “ VZV “ [Title/Abstract] OR “HZ” [Title/Abstract]
#3	#1 OR #2
#4	“ Minimal invasive surgery “ [Mesh] OR “ Nerve block “ [Mesh] OR “, Intrathecal drug injection “ [Mesh] OR “ Pulsed-radiofrequency “ [Mesh] OR “ MIS “ [Title/Abstract] OR “ Tiny traumatic interventional treatment “ [Title/Abstract] OR “ Microinvasive intervention “ [Title/Abstract] OR “ Minimally invasive technic “ [Title/Abstract] OR “ MIT” [Title/Abstract] OR “ AS-N “ [Title/Abstract] OR “ CPNB “ [Title/Abstract] OR “ Nerveblock “ [Title/Abstract] OR “ Intraspinal injection” [Title/Abstract] OR “ Pulsed RF” [Title/Abstract] OR “ PRF” [Title/Abstract] OR “ Pulse RF” [Title/Abstract]
#5	“Acupuncture” [Mesh] OR “Cupping” [Title/Abstract] OR “Moxibustion” [Title/Abstract] OR “Chinese External Medicine” [Title/Abstract] OR “Electroacupuncture” [Title/Abstract] OR “Fire Needle” [Title/Abstract] OR “Bloodletting” [Title/Abstract] OR “Acupuncture injection” [Title/Abstract] OR “Plum blossom needle” [Title/Abstract]
#6	#4 OR #5
#7	“Randomized Controlled Trial” [Title/Abstract] OR “Controlled Clinical Trial” [Title/Abstract] OR “Clinical Trial” [Title/Abstract] OR “Clinical Trial” [Publication Type] OR “RCT” [Title/Abstract]
#8	#3 AND #6 AND #7

HZ = herpes zoster, PHN = postherpetic neuralgia.

### 2.3. Data collection and analysis

#### 2.3.1. Study selection.

**Figure 1.**
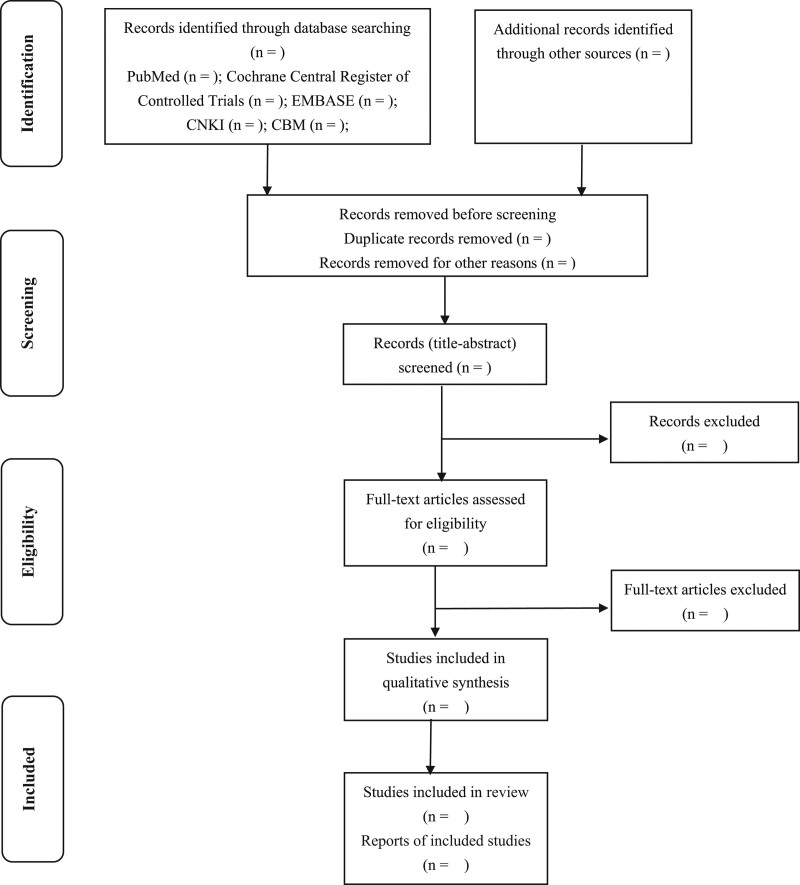
Flow diagram of the study selection process. Adapted from preferred reporting items for systematic reviews and meta-analysis protocols.

All reviewers will receive professional training to understand the objective and process of the review before the selection of studies. Literature search results will be imported into ENDNOTE X8 software. The duplicates will be removed. For studies that have been updated, the older 1 will be excluded, or can be used as supplementary data in further research. Titles and abstracts will be screened independently by 2 reviewers (ZQP and SJH). Full texts will be obtained for eligible studies and will be screened independently (ZQP and SJH). Discrepancies will be resolved through discussion, or by consulting a third reviewer (AZ). The procedures of study selection will be performed in accordance with the Preferred Reporting Items for Systematic reviews and Meta-Analysis flow chart (as shown in Fig. 1).

#### 2.3.2. Data extraction and analysis.

Two reviewers (ZQP and SJH) will establish a sheet using Microsoft Excel 2010, pilot and refine this form using 10 initial studies. After the form has been developed, the 2 reviewers will extract data from the text and figure/table independently, including: general information (e.g., author, year of publication, country where the study was conducted, study period, original inclusion criteria); participants (e.g., age, gender, sample size); details about intervention (e.g., surgical treatments type, administration method, duration of study, follow-up time, acupuncture parameters, acupuncture points); study design (e.g., randomization, blinding and allocation concealment); outcomes and adverse reactions.

Discrepancies will be identified and resolved through discussion (with a third reviewer where necessary), or by consulting a third reviewer (JW). We first try to extract numerical data from tables, text or figures. If these are not reported, we will extract data from graphs using digital ruler software. In case data is not reported or unclear, we will attempt to contact authors by e-mail (max. 2 attempts).

WinBUGS and Stata 15.1 will be used in data analysis.^[23]^ For dichotomous data, a risk ratio with 95% confidence intervals will be used for analysis. For continuous data, a mean difference or a standard mean difference with 95% confidence intervals will be used for analysis.

#### 2.3.3. Classification of arms.

Classification of arms will be carried out at the data extraction stage. The arms of each trial will be classified according to the type of acupuncture intervention, type of surgical treatments or type of control condition employed, where applicable. The type of surgical treatments included in this study are those used as first-line treatment, as indicated by the guidelines for PHN.^[5,21]^ The type of external therapies of TCM will be classified according to the different types reviewed by a published article^[20]^in their review of interventions for the treatment of PHN. These classifications will include ordinary acupuncture, electro-acupuncture, auricular acupuncture, acupuncture injection, moxibustion, bloodletting, cupping, fire needle, and plum blossom needle, which may include combinations of the any of the previously listed types of acupuncture intervention. Control conditions will be classified as active or passive. Passive control conditions are those where no intervention is applied, while active control conditions involve some intervention (e.g., placebo, usual care).

A network diagram will be generated to visualize the evidence available for analysis, both in terms of possible pairwise comparisons and the volume of evidence underlying each of these comparisons. The reference node will contain active control conditions. Different combinations of interventions will be included as separate nodes. A network diagram will be generated to visualize the evidence available for analysis, both in terms of possible pairwise comparisons and the volume of evidence underlying each of these comparisons.^[24]^ The reference node will contain active control conditions. Different combinations of interventions will be included as separate nodes.

#### 2.3.4. Risk of bias.

For studies which are passed through the full-text screening stage, risk of bias will be assessed independently by 2 reviewers (ZQP and SJH) using the standard Cochrane risk of bias tool in Covidence which assesses sequence generation and allocation concealment, blinding of participants and personnel, blinding of outcome assessors and incompleteness of the outcome data and whether reporting appears to be selective.^[25]^ Discrepancies will be identified and resolved by consulting a third reviewer (JW).

#### 2.3.5. Assessment of transitivity.

A table of trial characteristics which may act as effect modifiers will be compiled from the data collected (as specified above) to aid in the assessment of the assumption of transitivity. Potential effect modifiers include total study duration, setting, age distribution, gender distribution, ethnicity distribution, patient comorbidity history and past/present medication use.^[26,27]^

### 2.4. Data synthesis

The main objective of this data synthesis is to compare the effectiveness of different interventions focused on external therapies of traditional Chinese medicine and surgical treatments. Network meta-analysis is useful for achieving this objective because it allows indirect estimates to be computed where little direct evidence exists. Few studies have directly compared external therapies of traditional Chinese medicine and surgical treatments. Studies will be pooled according to the trial arm classifications noted above. If quantitatively pooling the study results is not possible, the findings of the systematic review will be described narratively.

#### 2.4.1. Pairwise meta-analysis.

Where head-to-head data is available, exploratory pairwise meta-analyses will be conducted. These will be run using a random-effects model. The individual and pooled effect sizes will be visualized using Forrest plots. Funnel plots and Egger’s test will be employed to examine publication bias and the effects of small studies^[28]^

#### 2.4.2. Network meta-analysis.

If the assumption of transitivity is deemed to be met, random-effects Network meta-analyses will be conducted within a Bayesian framework using vague priors. These analyses will be carried out in line with the framework set out by Dias et al^[29]^ Estimates of the pairwise comparison of each intervention in the network will be presented in tables in the final manuscript, as will rankings demonstrating the probability of each intervention producing the best outcome. These rankings will be presented with mean ranks, 95% credible intervals and the surface under the cumulative ranking curve. Convergence will be assessed by checking if the Gelman-Rubin statistic is less than 1.1.^[30,31]^

#### 2.4.3. Assessment of inconsistency, heterogeneity and quality of the evidence.

Statistical heterogeneity will be assessed for each pairwise meta-analysis using the *I*^2^ and *τ*^2^ statistics in line with the Cochrane guidelines. Since the included studies are likely to consist of a mixture of 2-arm and multi-arm studies, it is necessary to consider design inconsistency as well as loop inconsistency. This will be achieved by applying a design-by-treatment interaction model. If inconsistency is indicated in the network, any closed loops within the network will be assessed.^[32]^

The quality of the evidence used in this study will be assessed using the GRADE 4-step approach for rating the quality of treatment effect estimates from network meta-analysis.^[33]^

#### 2.4.4. Additional analyses.

Exploratory analyses will be carried out, where there is a sufficient amount of information available to do so. These analyses will focus on the covariates the age distribution, gender distribution and ethnicity distribution. Network meta-regressions will be conducted to individually examine the influence of these covariates on effect size estimates. Finally, sensitivity analyses will be conducted to assess the influence of the use of specific treatments in the network rather than classes and trial quality.

## 3. Discussion

With the aging of the population and people’s living habits, the incidence of PHN is on the rise.^[2,34,35]^ Meanwhile, PHN is notoriously difficult to treat; complete resolution of symptoms is rare, and only less than 50% of PHN patients can achieve noteworthy pain relief.^[36]^ PHN seriously disturbs the patient’s sleep, produce anxiety, depression and other adverse psychology; in further, PHN affects patient’s quality of life, and brings enormous economic pressure.^[37]^ Therefore, solving the treatment difficulties of PHN is beneficial to the development of mankind’s health career.

We hope that through this study, direct and indirect evidence on the efficacy of external therapies of traditional Chinese medicine and surgical treatments for the pain management of PHN will be found, and the rankings will be provided by using a network meta-analysis. This study aims to provide reliable evidence-based information for medical practitioners to choose different therapeutic strategies in the clinical treatment of PHN; at the same time, this study is also helpful for the design treatment guidelines and the adjustment of health insurance plans.

## Author contributions

**Conceptualization:** Zhengqi Pan, Shijie Huang.

**Data curation:** Zhengqi Pan, Shijie Huang, Tingting Ma.

**Formal analysis:** Zhengqi Pan, Shijie Huang

**Investigation:** Jie Wu, Tingting Ma.

**Methodology:** Jie Wu.

**Project administration:** Zhengqi Pan.

**Resources:** Rongli Yuan, Rui Luo.

**Software:** Rongli Yuan, Mengjing Wang, Tingting Ma.

**Supervision:** Jie Wu.

**Validation:** Mengjing Wang, Maogui Yu, Wuyu Li, Ao Zhang.

**Writing – original draft:** Zhengqi Pan, Shijie Huang.

**Writing – review & editing:** Zhengqi Pan, Shijie Huang.

## Contributorship statement

I certify that neither this manuscript nor 1 with substantially similar content under my authorship has been published or is being considered for publication elsewhere (except as indicated in an attachment). I have access to any data upon which the manuscript is based and will provide such data upon request to the editors or their assignees.
